# Bilateral intracochlear hemorrhage: A rare onset of chronic myelogenous leukemia

**DOI:** 10.1002/ccr3.8741

**Published:** 2024-04-20

**Authors:** Babak Flahat, Åsa Bonnard, Julia Arebro

**Affiliations:** ^1^ Department of Neuroradiology Karolinska University Hospital Stockholm Sweden; ^2^ Division of Otorhinolaryngology, Department of Clinical Science, Intervention and Technology Karolinska Institutet Stockholm Sweden; ^3^ Department of Otorhinolaryngology, Head and Neck Surgery Karolinska University Hospital Stockholm Sweden

**Keywords:** ear, hematology, nose, throat

## Abstract

Acute onset of vertigo and hearing loss is rare in leukemic disorders. MRI can diagnose intracochlear hemorrhage as the underlying cause. The hearing can improve but if severe hearing loss preserves, cochlear implantation can be considered.

## 1 INTRODUCTION

Vertigo and severe hearing loss as debut of hematological diseases like chronic myelogenous leukemia (CML) is uncommon. Intracochlear hemorrhage is one possible cause and can be diagnosed with magnetic resonance imaging (MRI).^1^, ^2^ CML is nowadays successfully treated with tyrosine kinase inhibitors (TKIs) and the medication is generally well tolerated by the patients. Rare pulmonary complications have however been described, but seldom outside Asia.^3^, ^4^, ^5^, ^6^, ^7^ We here present an unusual case with bilateral intracochlear hemorrhage at onset of CML, with the rare complication of pneumonitis, and lung fibrosis secondary to treatment with the TKI Imatinib. The hearing loss secondary to the intracochlear hemorrhage is often permanent but, in this case, one side improved over 6 months. If severe hearing loss remained, cochlear implantation would have been considered.^1^, ^8^, ^9^

## 2 CASE HISTORY

A 77‐year‐old man with previous normal hearing, suddenly suffered from heart burn and vertigo. The patient's medical history involved hypertension and heart attack, treated twice with angioplasty and stent. His current medication included calcium blocker, ACE inhibitor, and acetylsalicylic acid. At examination, a big spleen was noticed but no neurological deficit was present. Laboratory examination of peripheral blood revealed a leukocyte count of 553 × 10^9^/L with 13.3 × 10^9^L blasts, 125, 5 × 10^9^/L myeloid cells, 516 × 10^9^/L platelets, and a hemoglobin level of 86 g/L (Table 1). CML was suspected due to the typical differential blood count and leukapheresis was started the same day followed administration of Hydroxycarbamide and Imatinib medication. The following day, the vertigo had increased along with an onset of hearing loss and tinnitus on his right ear. Neurological examination revealed a spontaneous left horizontal nystagmus and a positive head‐impulse test to the right side. Hearing loss on the left side followed a couple of days later.

**TABLE 1:** Laboratory examination of peripheral blood at onset of vertigo and CML.

Cell type	Concentration
B‐Leucocytes	553 × 10^9^/L
B‐Blast cells	13.3 × 10^9^/L
B‐Basophil granulocytes	14.4 × 10^9^/L
B‐Eosinophil granulocytes	9.4 × 10^9^/L
B‐Neutrophil granulocytes	300.3 × 10^9^/L
B‐Monocytes	15.5 × 10^9^/L
B‐Lymphocytes	5.5 × 10^9^/L
B‐Myelocytes	125.5 × 10^9^/L
B‐Promyelocytes	249 × 10^9^/L
B‐Metamyelocytes	44.2 × 10^9^/L
B‐Erythroblasts	5.5 × 10^9^/L
B‐Hemoglobin	86 g/L
B‐Trombocytes	516 × 10^9^/L

## 3 METHODS

**FIGURE 1:**
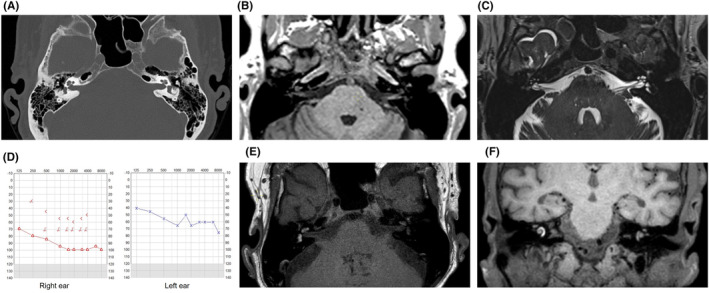
(A) CT scan of the ear without any inflammatory or neoplastic changes in the middle ear and normal otic capsule. (B) Pre‐contrast axial T1‐weighted MRI imaging showing a slight hyperintensity in the area of the cochlea/vestibular apparatus on the right side and in the cochlea on the left side due to the presence of methemoglobin from blood. (C) T2‐weighted MRI imaging showing loss of perilymph signal mainly in the scala tympani and in the vestibule in the right cochlea. (D) Tone audiogram on day 7. (E, F) Follow up pre‐contrast axial T1‐weighted MRI and fat saturated coronal images showing even more distinct signs of bilateral inner ear hemorrhage.

Repeated CT scans showed no pathology of the middle or inner ears (Figure 1A), but an MRI revealed bilateral intracochlear hemorrhage most prominent on the right side (Figure 1B,C). An audiogram showed nearly total loss of hearing on the right ear and moderate to severe hearing loss on the left ear (Figure 1D). The patient underwent a seven‐day treatment of Dexamethasone 20 mg/day followed by 10 days with a decreasing dose combined with vestibular physiotherapy. The vertigo decreased the following days, but a follow up MRI revealed even more distinct signs of bilateral inner ear hemorrhage (Figure 1E,F). A control audiogram was performed 8 days later revealing aggravation in the right ear, with hearing threshold at 108 dB, but a slightly better result on the left side (Figure S1). Head‐impulse test was now bilateral pathological and vHIT confirmed bilateral impairment of vestibular function. The vertigo markedly decreased 10 days after onset, but since the patient experienced great problems with the hearing loss, a potential cochlear implant (CI) was investigated.

Examination of the bone marrow and peripheral smear (Figure S2A–G) along with fluorescence in situ hybridization (FISH) revealing BCR‐ABL in 99% of cells established the diagnosis of chronic myeloid leukemia. Five weeks after onset of symptoms, a follow up examination was performed with vestibular‐evoked myogenic potential (VEMP). The patient's vertigo had decreased but VEMP indicated bilateral reduced function and head‐impulse test was still pathological bilaterally. Tonal audiogram showed improved hearing on the left side and possibly even on the right side (Figure S3).

The same day, the patient experienced dyspnea, low blood oxygen level and fever. Initially the patient was considered to have a pneumonia and a CT scan verified bilateral pulmonary embolism in addition. The patient received adequate medication and the infection and embolism seemed under control, however, the patient's dyspnea increased. A new CT scan found ground glass attenuations in bilateral lungs (Figure S4). Finally, the patient was diagnosed with Imatinib related pneumonitis followed by pulmonary fibrosis since no cause other than Imatinib was found. Imatinib was terminated and instead, Hydroxycarbamide and Bosutinib was initiated. For the parenchymal changes and pulmonary symptoms, the patient was treated with Betamethasone 6 mg/day followed by at least 6 months with decreasing dose, with improvement of the symptoms. Ten days after diagnosis of Imatinib induced pneumonitis, the patient suffered from upper abdominal pain diagnosed with coronary angiography as lateral ST‐elevation myocardial infarction (STEMI) and was treated with acetylsalicylic acid and Clopidogrel.

## 4 CONCLUSION AND RESULTS

**FIGURE 2:**
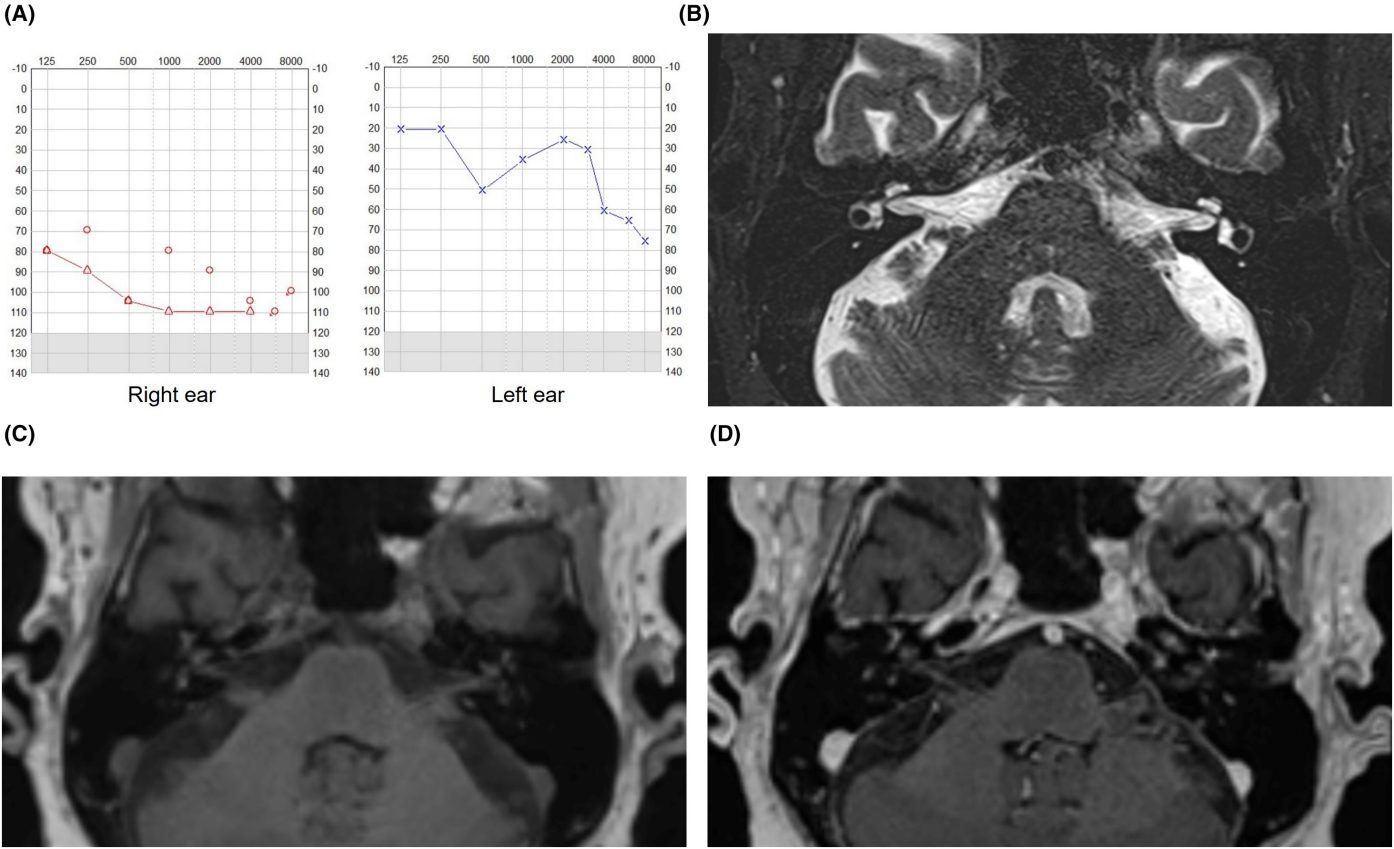
(A) Tone audiogram 6 months after debut of inner ear symptoms. (B) T2‐weighted MRI imaging revealing normalization of perilymph signal in the cochlea. (C, D) T1‐weighted MRI imaging before and after intravenous gadolinium contrast medium showing normalization of perilymph signal in the cochlea and in the vestibular apparatus. No sign of pathological contrast enhancement in the labyrinth.

The patient was rehabilitated and experienced over all successively less vertigo the following 4 month. The hearing improved (Figure 2A), enabling conversations in between up to six people. Speech perception on the left ear without hearing aids was 80% at 70 dB, while the quality of hearing was too bad on the right side for testing. However, situations with more people than six was still challenging for the patient, especially if involving children, and some tinnitus remained. An MRI confirmed resorption of the vestibulocochlear hemorrhage and ruled out any fibrotization or ossification in the labyrinth (Figure 2B–D). The need for CI was paused temporarily since an CI for unilateral hearing loss is just indicated in cases with severe tinnitus in Sweden. This case illustrates that hematological disorders should be considered in the differential diagnosis of sudden sensorineural hearing loss, and that intracochlear hemorrhage is diagnosed with MRI. The case demonstrates the importance of follow‐up to consider cochlear implantation.

## 5 DISCUSSION

Vertigo and sudden hearing loss as primary symptoms of leukemia are rare. Few cases with inner ear symptoms at onset of CML have been reported worldwide.^10^, ^11^, ^12^, ^13^, ^14^, ^15^, ^16^ The inner ear symptoms can be explained by hemorrhage, like in this case, or by leukemic infiltration such as hyperleukostas or leukemic infiltration, or by infection.^10^ Confirmed bilateral intracochlear hemorrhage at onset of CML has previously just been described in one case to our knowledge.^17^ Using both T1 as well as T2‐weighted MRI imaging, Yang et al. diagnosed a 25‐year‐old man recently diagnosed with CML to have bilateral intracochlear hemorrhage. The symptoms of tinnitus and hearing loss was bilateral but asymmetrical with profound hearing loss on one side and a moderate hearing loss on the other side. MRI findings correlated to the asymmetrical symptoms but unfortunately, the case report does not report follow‐up on patients' symptoms.^17^ The prognosis of profound hearing loss due to intracochlear hemorrhage (also referred to as intralabyrinthine hemorrhage) is generally known to be poor. Cochlear implantation 4 month after onset of inner ear hemorrhage symptoms has been described with successful results in a patient with a past medical history of myelomonocytic chronic leukemia (CMML).^8^ Several cases with successful cochlear implantation after intracochlear hemorrhage without hematologic disorders has also been described.^1^, ^9^ As described by Kaya et al., intracochlear hemorrhage does not damage inner hair cells, spiral ganglion cells, the stria vascularis, or the spiral ligament, making cochlear implantation possible on a cellular level if needed.^18^ Cochlear fibrosis and ossification must however be excluded before surgery since it may develop within a couple of month.^19^, ^20^ Ryu et al. studied the effects of intracochlear bleeding during cochleostomy in cochlear inflammatory response and residual hearing in a guinea pig animal model.^19^ More extensive fibrosis and ossification was observed in blood injected ears compared to normal ears or cochleostomy only ears.

CML, also known as chronic myeloid leukemia, is a myeloproliferative disorder characterized by presence of the Philadelphia chromosome resulting in increased and unregulated production of granulocytes and their precursors. As a result, blood, bone marrow, and tissues are infiltrated by neoplastic cells of granulocyte lineage. The entrance of TKIs has dramatically improved the treatment of patients with CML. Imatinib was the first TKI for the treatment of CML. It was approved in 2001 and has been followed by second and third generation TKIs.^21^ Imatinib is in general well tolerated by patients and adverse events can be divided into hematological (anemia, neutropenia, thrombocytopenia) vs nonhematological (cardiovascular, pancreatic, hepatic, cutaneous, gastrointestinal effects; fluid retention, bleeding, musculoskeletal disorders, neuropathy, ocular toxicity, infectious, or metabolic events). Cases with pneumonitis and pulmonary interstitial fibrosis like in the case here described have been reported previously^3^, ^4^, ^5^ but rarely outside Asia.^6^, ^7^ Several authors have reported on succesful outcome of pulmonary adverse events upon discontinuation of imatinib in combination with initiation of corticosteroid treatment.^3^, ^4^, ^5^, ^6^, ^7^ Ohnishi et al. however reported on 27 patients with Imatinib‐induced interstitial lung disease.^4^ Imatinib was discontinuated in all the reported cases and 24 cases were treated with corticosteroids, however, four patients failed to improve and one patient suffered from pulmonary fibrosis despite high‐dose corticosteroid treatment.^4^ Imatinib‐induced pulmonary lung disease with possible irreversible outcome must be taken into account in patient management.

Inner ear hemorrhage in patients with leukemia has been described since 1884 by Politzer, around 100 years before the development of MRI as a medical imaging technique.^22^ Today, inner ear MRI represents gold standard for diagnosis and should be considered when sudden sensorineural hearing loss is present along with suspected or confirmed hematological disease.^1^, ^2^ Chen et al. examined the inner ears of 1252 patients with sudden sensorineural hearing loss with MRI. As many as 24 patients (1.9%) were diagnosed with intralabyrinthine hemorrhage. All patients with intralabyrinthine hemorrhage experienced unilateral hearing loss with profound deafness in 79.2% of cases. Vertigo was present in 95.8% and tinnitus in 79.2% of cases. A treatment protocol was applied to all 1252 patients and out of the 24 patients with intralabyrinthine hemorrhage, 8/24 showed improved hearing over time. None of the patients was however diagnosed with leukemia. All 24 patients underwent a follow up MRI demonstrating absorbance of the intracochlear hemorrhage within 2 weeks to 4 months. This illustrates the importance of MRI as a diagnostic tool in selected cases of sudden hearing loss, and also reveals the natural course of the condition in terms of imaging and symptomatology.

To summarize, vertigo and hearing loss as only symptoms is a rare onset of CML. We recommend MRI to diagnose a possible intracochlear hemorrhage. The case presented here, with bilateral hemorrhage, clearly reveals that the hearing loss can improve but it can also cause permanent symptoms despite resorption of the hemorrhage.

## AUTHOR CONTRIBUTIONS

**Babak Flahat:** Methodology; visualization; writing – review and editing. **Åsa Bonnard:** Visualization; writing – review and editing. **Julia Arebro:** Conceptualization; formal analysis; funding acquisition; investigation; methodology; project administration; supervision; visualization; writing – original draft; writing – review and editing.

## FUNDING INFORMATION

This work was supported by grants from Karolinska Institutet, Karolinska University Hospital, the Acta Oto‐Laryngologica Foundation, and the Swedish Association for Otorhinolaryngology Head and Neck Surgery (SFOHH).

## CONFLICT OF INTEREST STATEMENT

The authors declare no conflicts of interest.

## CONSENT

Written informed consent upon review was obtained from the patient for the publication of this case report.

## Supporting information


Figure S1.

## Data Availability

The authors confirm that the data supporting the findings of this study are available within the article or in its supplementary materials.
