# Supplementary material for: Bilateral intracochlear hemorrhage: A rare onset of chronic myelogenous leukemia
Source: Clin Case Rep. 2024 Apr 20;12(4):e8741. doi: 10.1002/ccr3.8741 (PMC11031744; doi:10.1002/ccr3.8741)
Supplement: Supplementary file 1 — Figure S1. [file CCR3-12-e8741-s001.docx]

**Supplementary information**


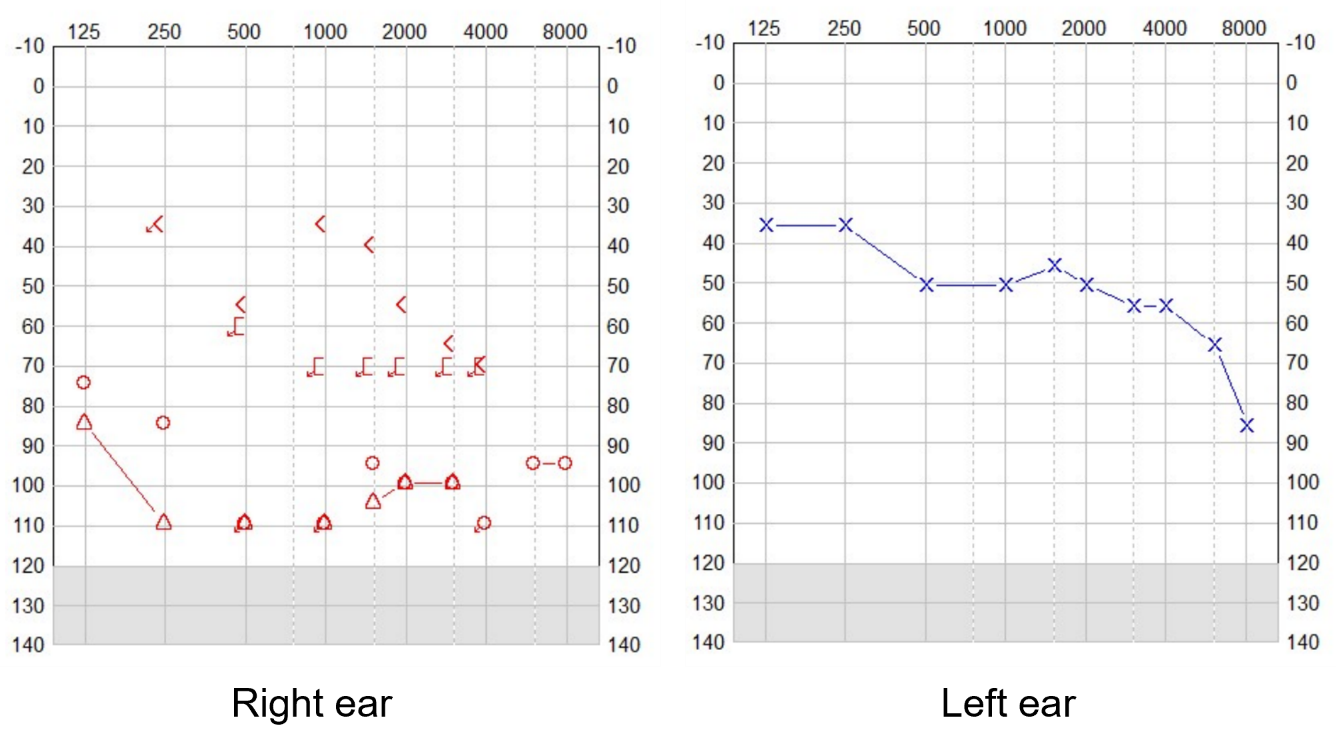


**Supplementary Figure 1.** Tone audiogram on day 15.


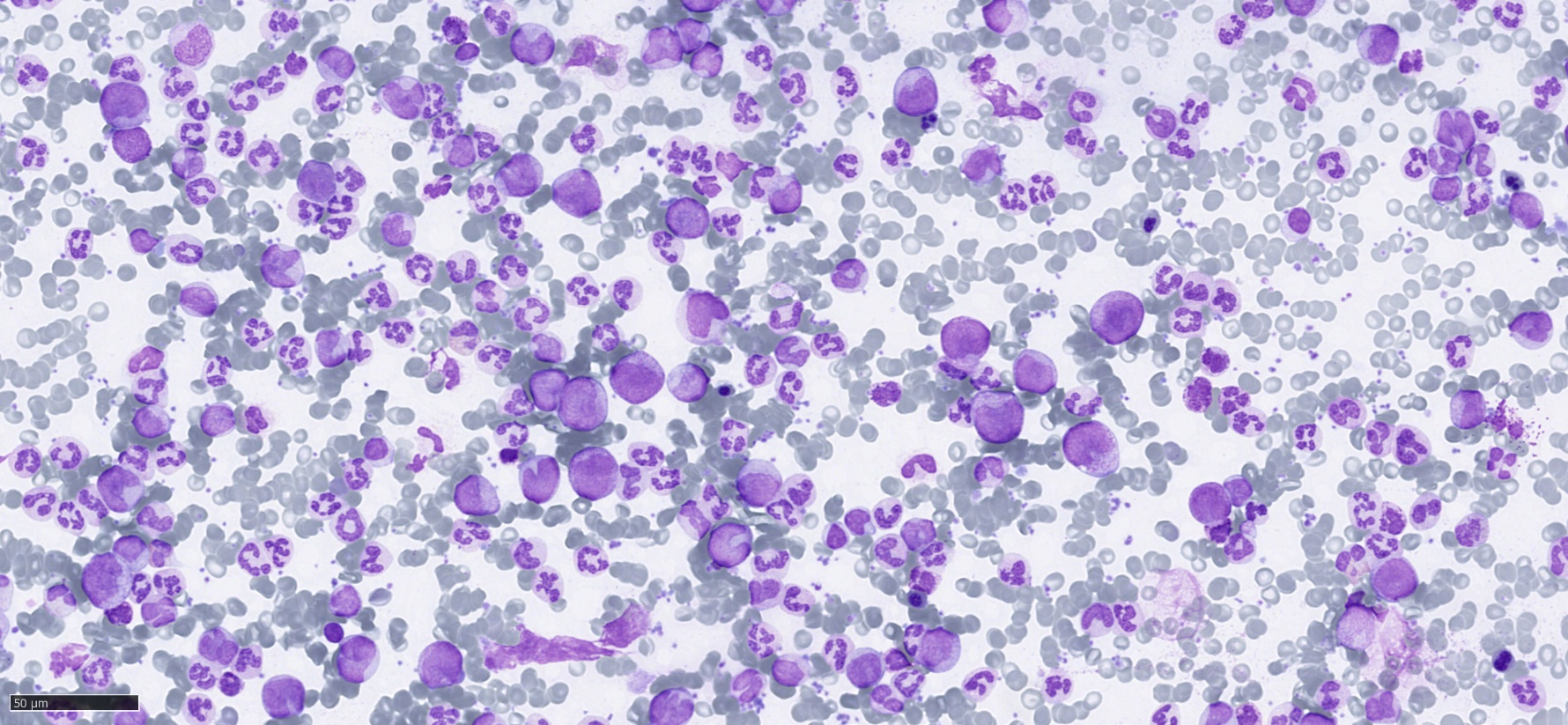

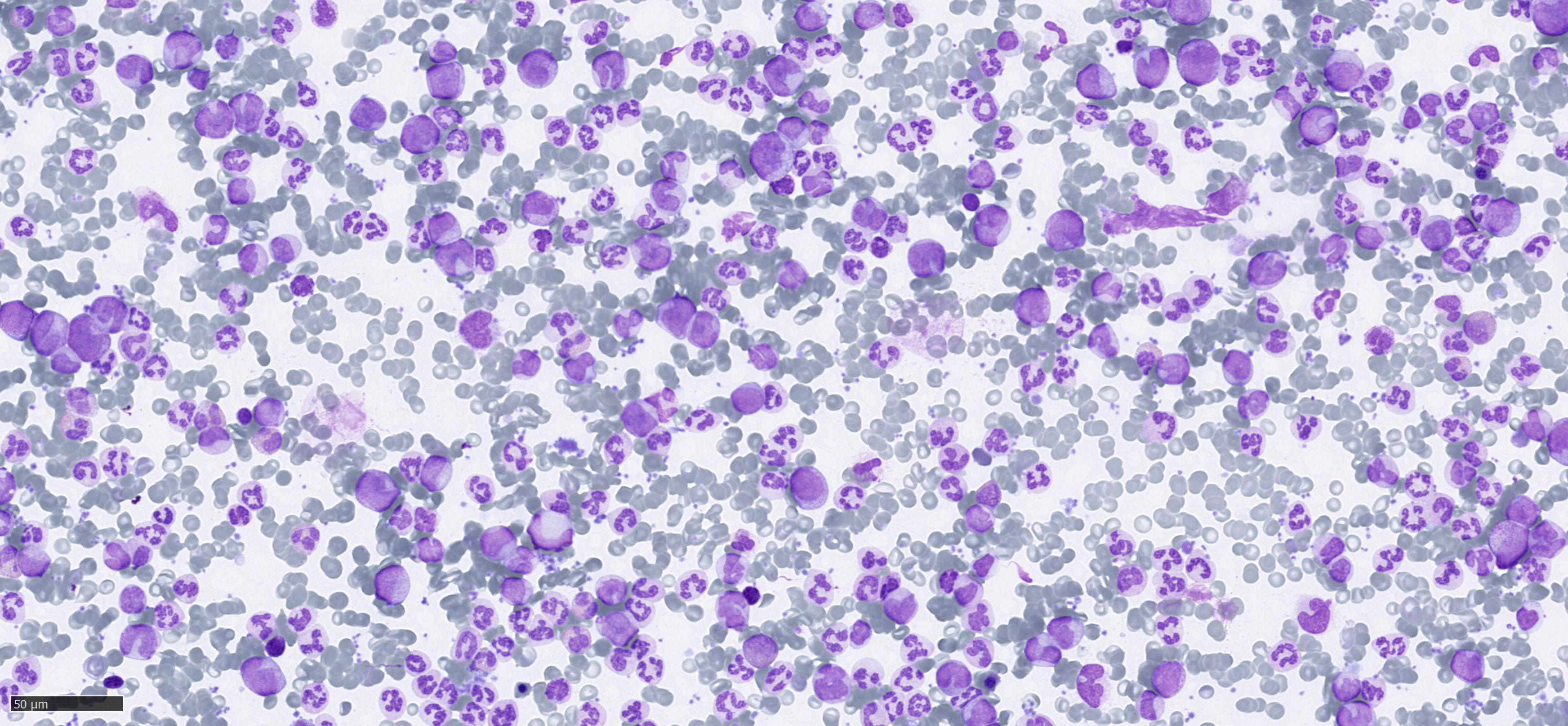

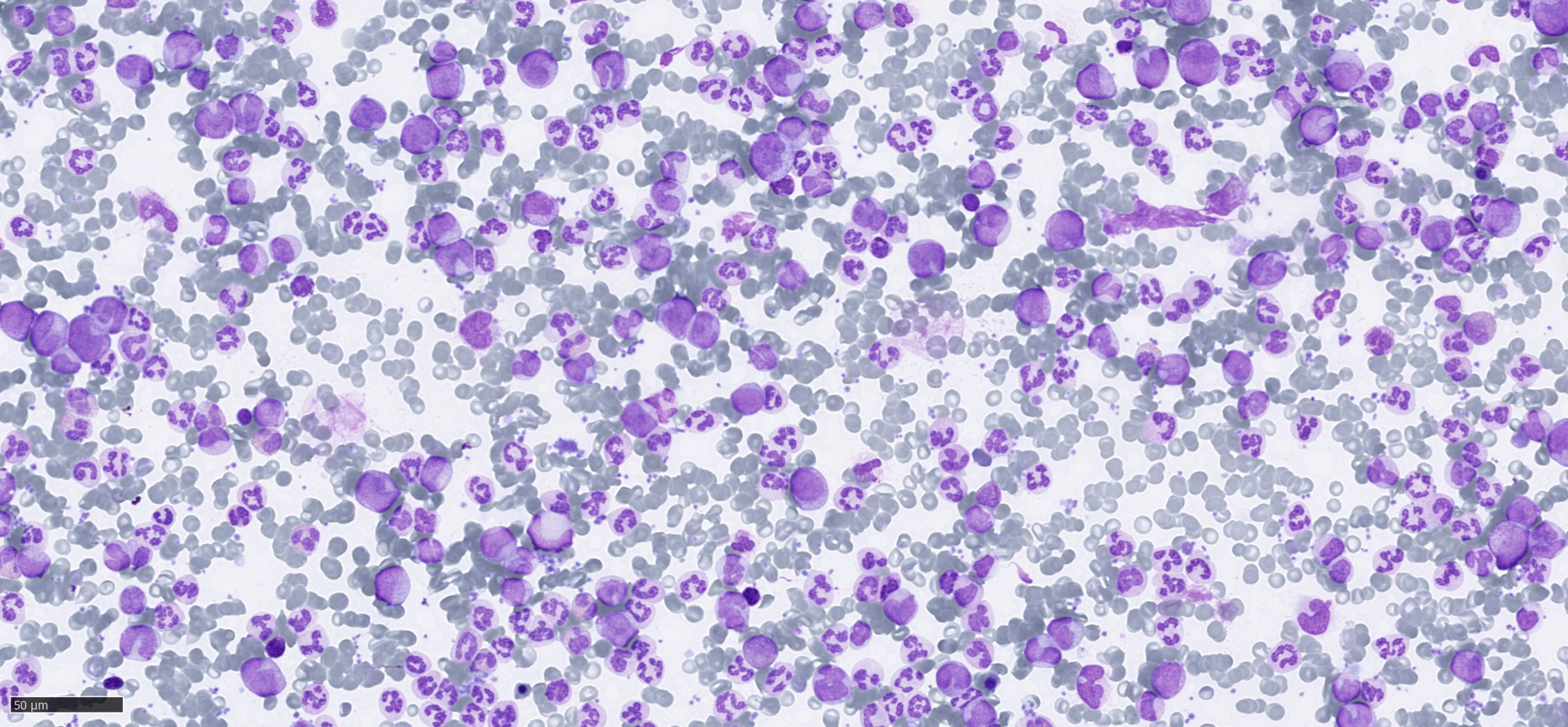

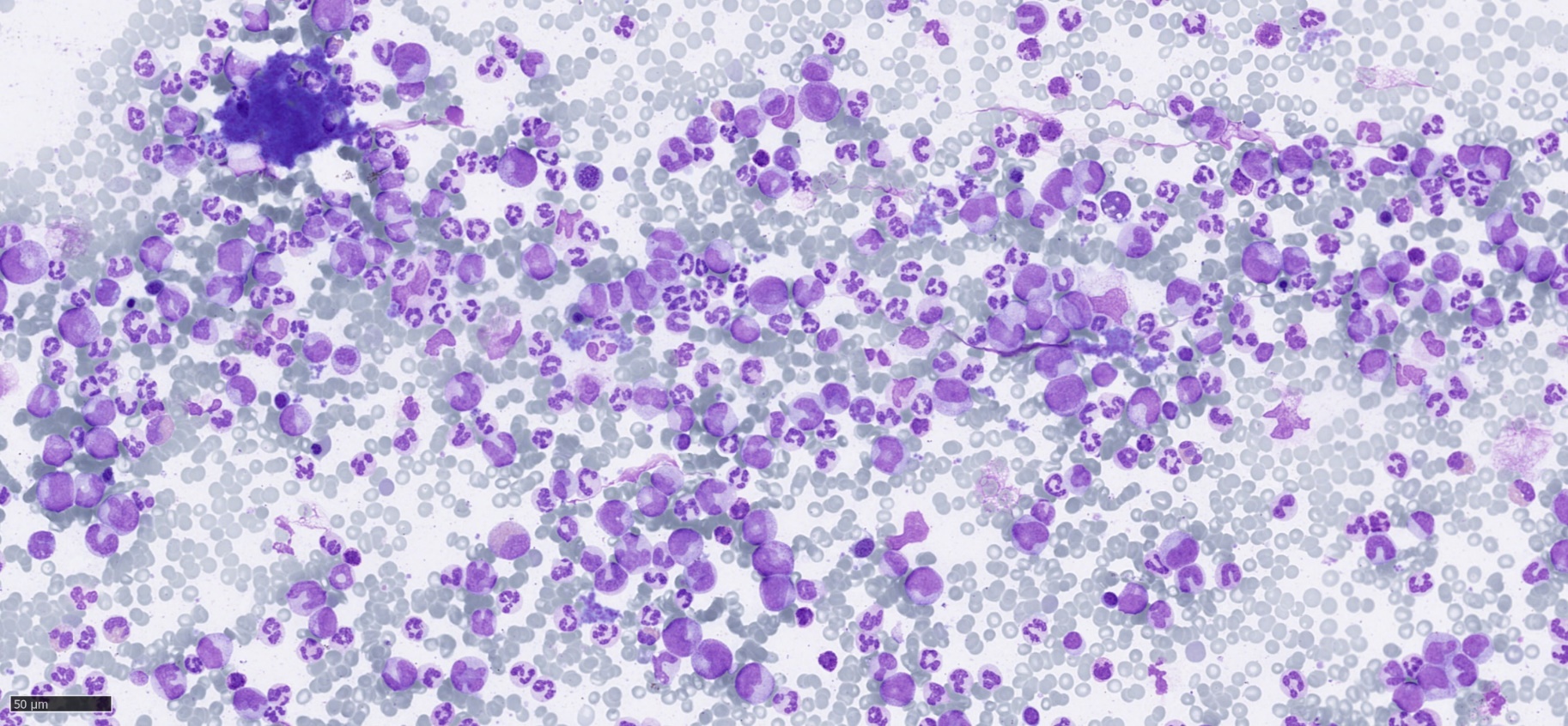

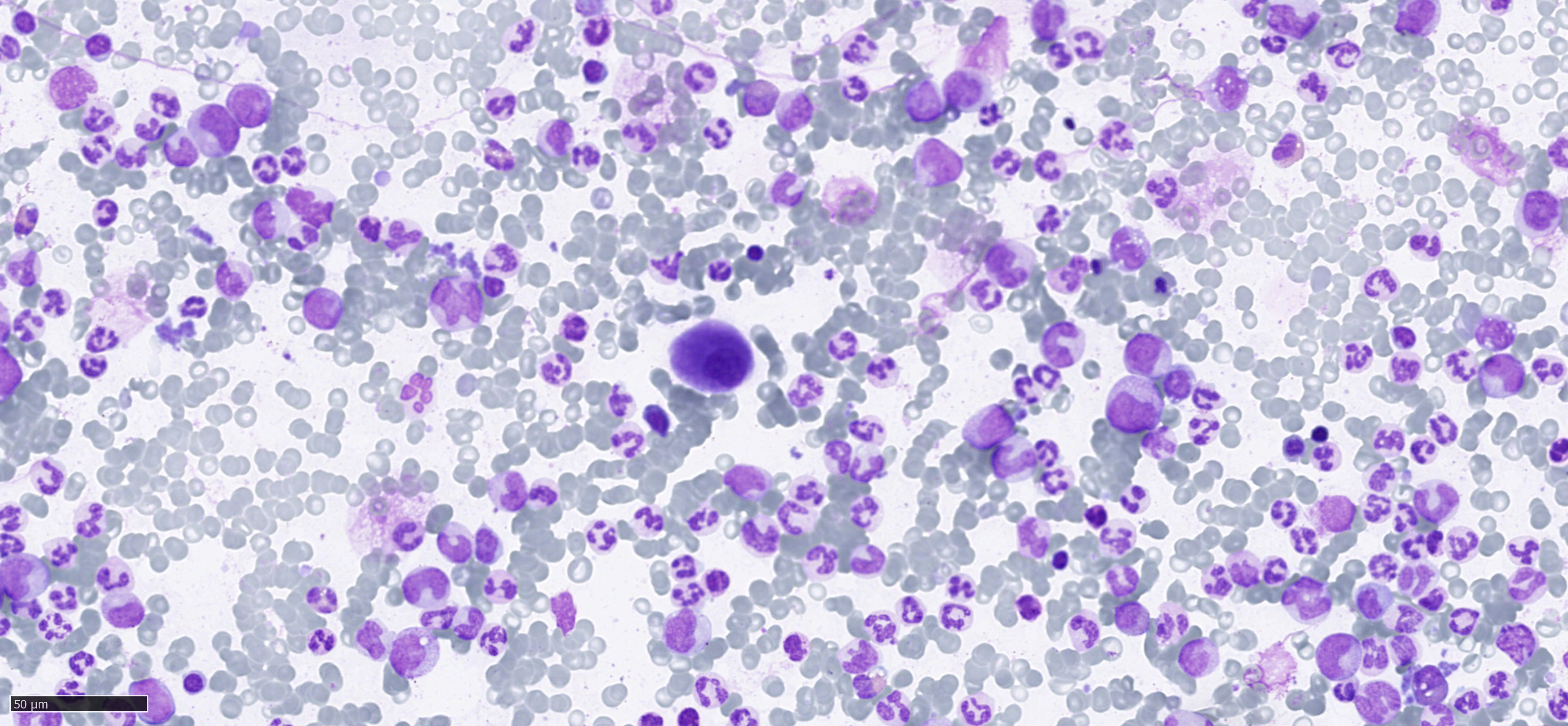

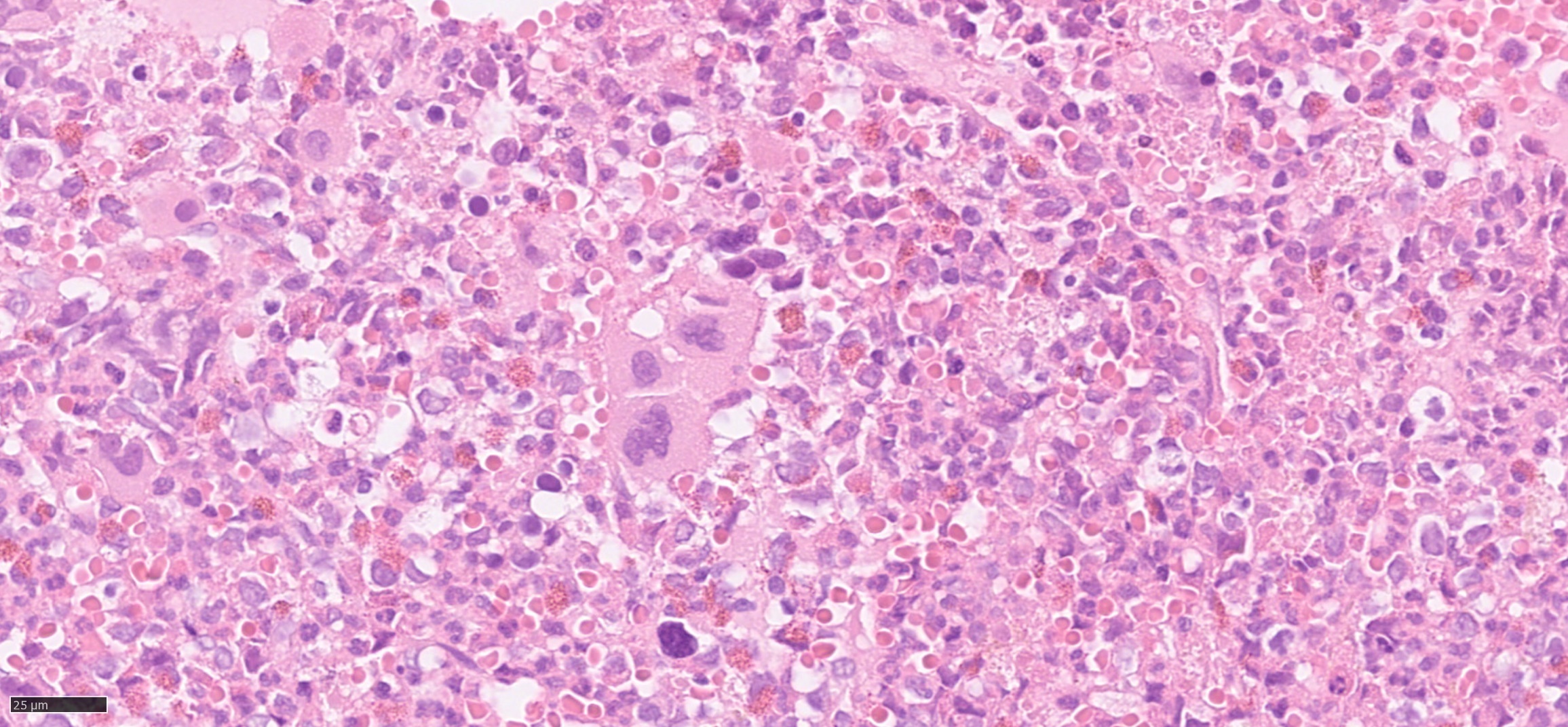

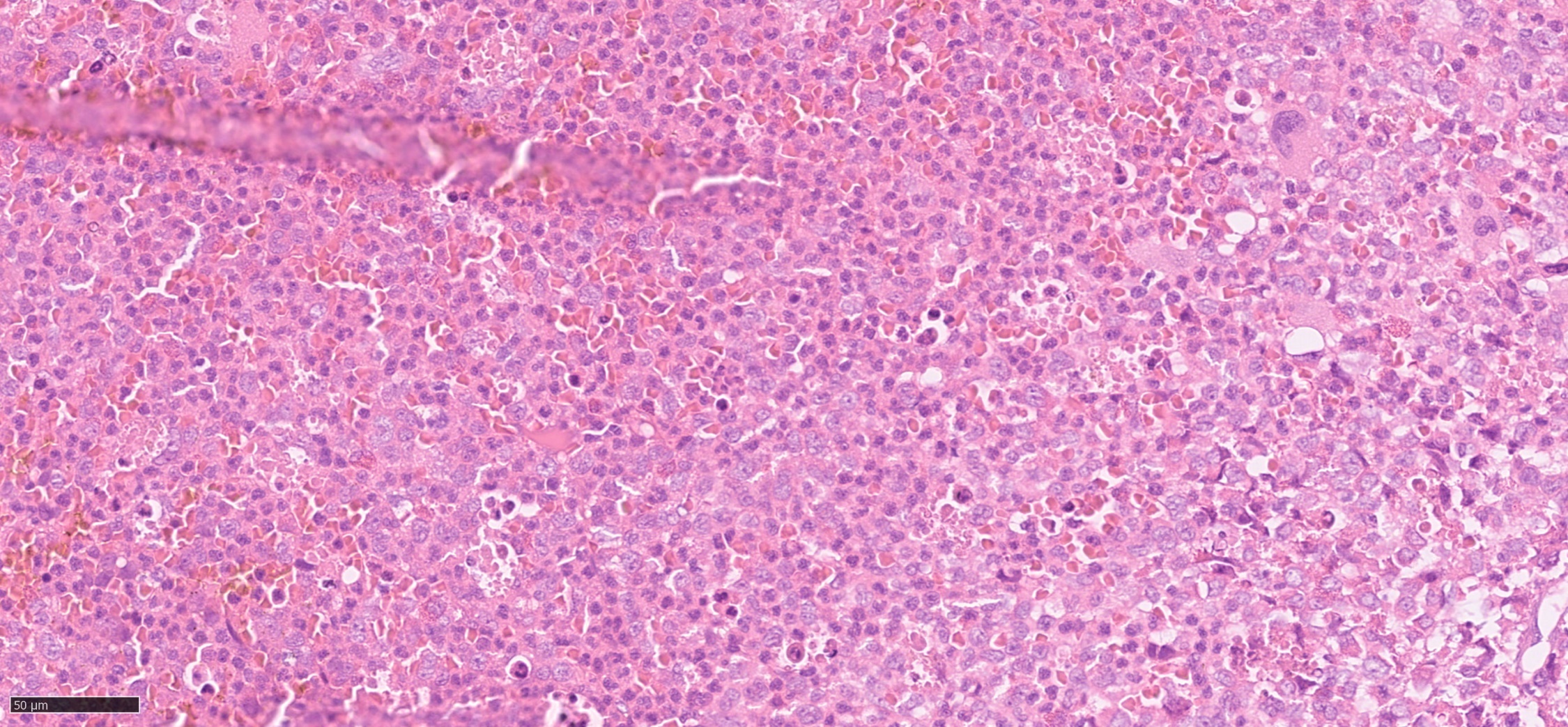


(e)

(g)

(f)

(d)

(c)

(b)

(a)

**Supplementary Figure 2.** Bone marrow biopsy with hematoxylin-eosin staining revealing (a) myeloid hyperplasia and (b), megakaryocytic hyperplasia. Bone marrow smear with May-Grünwald-Giemza staining showing (c) expansion of the myeloid cell line, and (d) bilobed, hyposegmented megakaryocyte. Peripheral blood smear with May-Grünwald-Giemza staining displaying (e) hyperleukocytosis and neutrophilia, (f) myeloblast (black star), and (g) promyelocyte (white star).

-


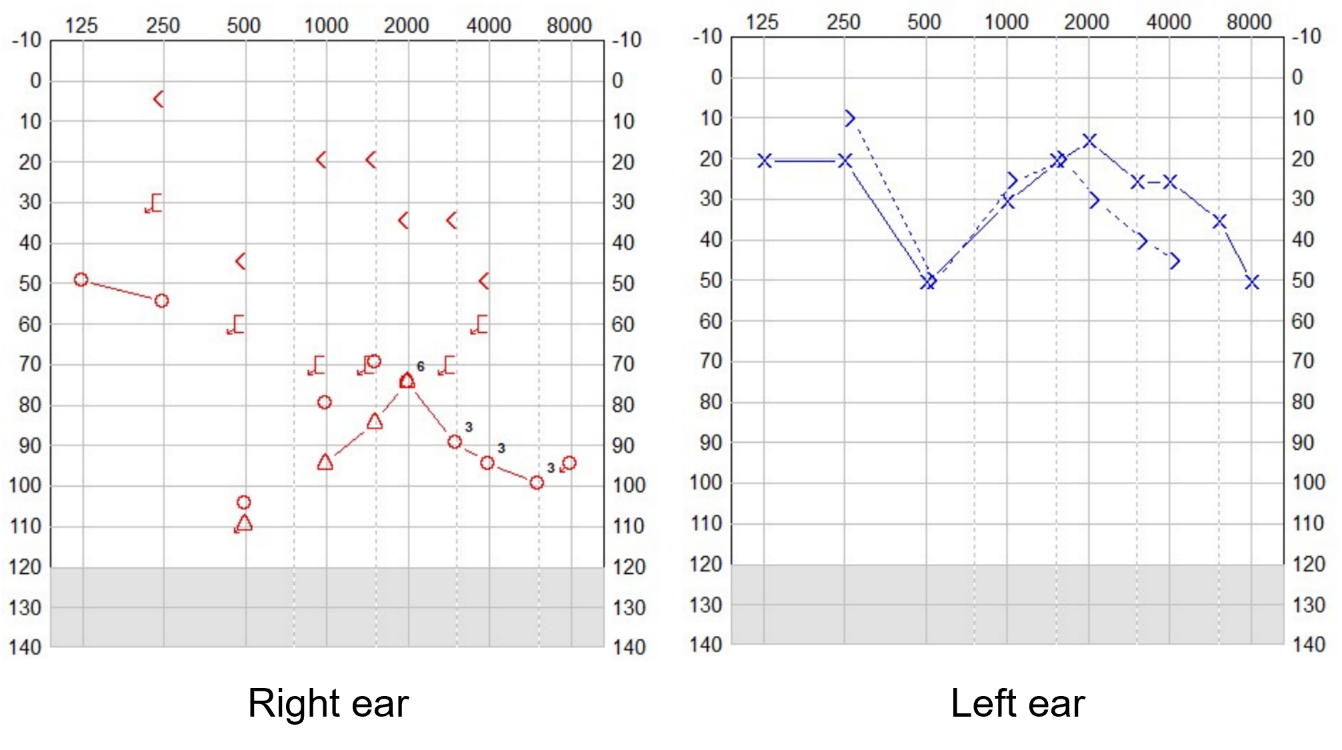


**Supplementary Figure 3.** Tone audiogram 3.5 months after debut of inner ear symptoms.


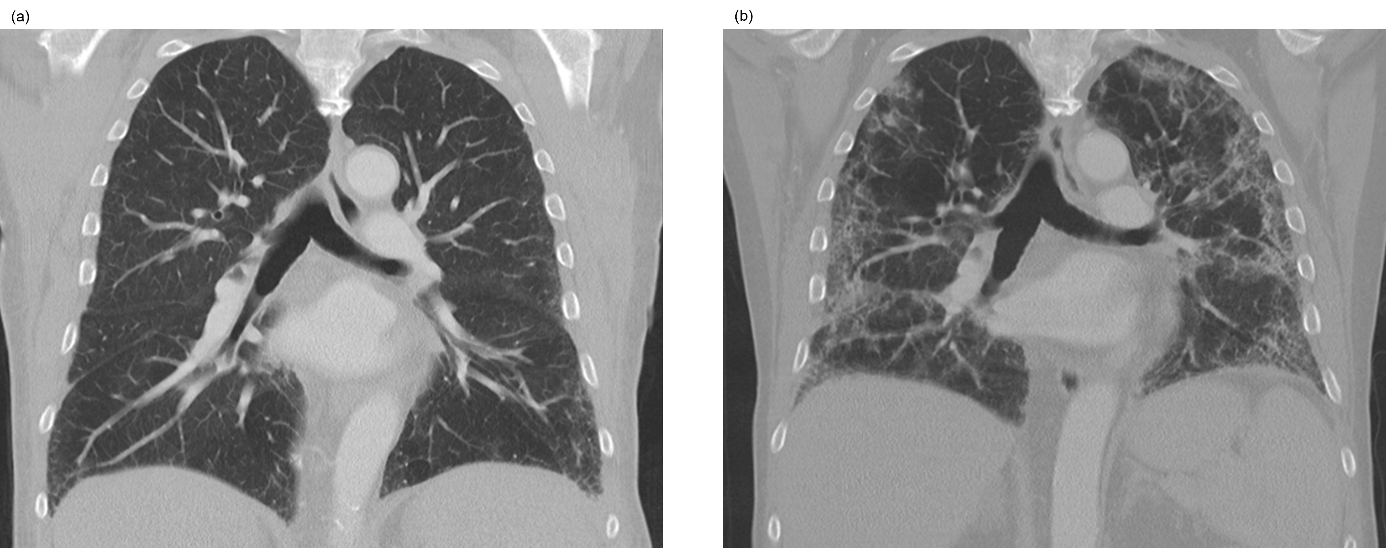


**Supplementary Figure 4.** (a) CT pulmonary angiography (CTPA) at onset of CML without significant findings. (b) CTPA five to six weeks after onset of CML and treatment start of Imatinib revealing pulmonary embolism (not shown here) and bilateral subpleural reticular and ground-glass opacities without basal dominance.
